# Evaluation of dynamic changes in interstitial fluid proteome following microdialysis probe insertion trauma in trapezius muscle of healthy women

**DOI:** 10.1038/srep43512

**Published:** 2017-03-07

**Authors:** Maria V. Turkina, Nazdar Ghafouri, Björn Gerdle, Bijar Ghafouri

**Affiliations:** 1 Division of Cell Biology, Department of Clinical and Experimental Medicine, Linkoping University, Sweden; 2 Pain and Rehabilitation Centre, and Department of Medical and Health Sciences, Linköping University, Linköping, Sweden

## Abstract

Microdialysis (MD) has been shown to be a promising technique for sampling of biomarkers. Implantation of MD probe causes an acute tissue trauma and provokes innate response cascades. In order to normalize tissue a two hours equilibration period for analysis of small molecules has been reported previously. However, how the proteome profile changes due to this acute trauma has yet to be fully understood. To characterize the early proteome events induced by this trauma we compared proteome in muscle dialysate collected during the equilibration period with two hours later in “post-trauma”. Samples were collected from healthy females using a 100 kDa MW cut off membrane and analyzed by high sensitive liquid chromatography tandem mass spectrometry. Proteins involved in stress response, immune system processes, inflammatory responses and nociception from extracellular and intracellular fluid spaces were identified. Sixteen proteins were found to be differentially abundant in samples collected during first two hours in comparison to “post-trauma”. Our data suggests that microdialysis in combination with mass spectrometry may provide potentially new insights into the interstitial proteome of trapezius muscle, yet should be further adjusted for biomarker discovery and diagnostics. Moreover, MD proteome alterations in response to catheter injury may reflect individual innate reactivity.

Microdialysis (MD) is an established technique for the *in vivo* sampling of various substances from the extracellular space of various tissues giving the possibility to monitor localized molecular events in tissues before changes occurs on the blood level. MD of muscle tissues has been used by our group to study nociceptive and metabolic mechanisms in different chronic musculoskeletal pain states. The exact nociceptive/inflammatory and neuropathic pain mechanisms underlying chronic muscle pain are very complex and not fully clarified^1^ and identification of myalgia “signatures” requires a detection method that allows sensitive, specific and reproducible identification, and quantification of potential biomarkers within a high dynamic range. During MD, a catheter with a porous membrane is implanted into the muscle, which is aimed to mimic the functions of a capillary blood vessel, where molecules from extracellular space are diffusing to the physiological saline (perfusion) solution along the concentration gradient and can be collected for following analyses. MD has been initially developed for sampling of rather small molecules (metabolites, amino acids, energy substrates, drugs and neurotransmitters). During the last decade the high molecular weight cut-off MD has demonstrated to be a promising technique for the sampling of protein biomarkers; however this application is rather complex in comparison to small molecule MD. To turn this technique into the robust and reliable daily clinical routine method for *in situ* monitoring of protein biomarkers, and to explore new clinically relevant biomarkers a deeper method understanding and optimization is needed.

MD causes minimal discomfort to patients and due to the small size of the probe is called a minimally-invasive procedure^2^. Nevertheless, the insertion of MD catheter induces mechanical damage of the surrounding tissue and blood vessels^3^,^4^,^5^, which results in local blood flow interruption and bleeding^5^,^6^,^7^, followed by cascades of fast signaling molecular events^6^. MD probe implantation itself can lead to inflammatory responses, both acute and chronic^6^, leading to a variety of substances being released from the damaged tissue, including inflammatory mediators, such as chemokines and cytokines. MD injury can also led to activation of sensory receptors including nociceptors if trauma is localized in the vicinity to them, generating sensation of pain^8^.

Therefore, there are reasonable concerns about inaccurate interpretation of rather complex stress, wound, wound repair, and disease response events: the disease-related and catheter injury-associated events can involve the same proteins/molecules and thus the sum of events, which can, potentially, compromise study outcomes^6^.

In order to minimize the influence of needle trauma on experimental outcomes, the “recovery and equilibration” period is often introduced to allow the vascular reaction to return to the normal (or stabilized) state^9^. Thus an equilibration period of two hours after probe injection is typically used in small molecular MD sampling protocols, and initial fractions under 2 hours often are discarded or not analyzed. The same two hours equilibration period is often used even in case of protein biomarkers sampling^10^,^11^,^12^, thus discarding potentially useful information about the individual innate response, which might be different depending on individual subject condition^3^. In this respect a better understanding of processes that occur in tissues during microdialysis is needed.

The present study aimed to evaluate changes in the proteome over time following catheter insertion in the trapezius muscle of healthy subjects. We performed proteomic analyses of MD fraction collected immediately after insertion of MD probe (trauma protein fraction (T)) and compared it to the fractions collected 2 hours later (post trauma fraction (PT)).

## Results and Discussion

In present study we used advantages of high-resolution mass spectrometry for the analysis of peptides obtained from two different muscle dialysate fractions of individual subjects: equilibration period “trauma” fraction (T), collected immediately after catheter insertion and a “post-trauma” fraction (PT) collected 2 hours later. Proteins from dialysate fractions obtained from trapezius muscle interstitium of six healthy female subjects were subjected to minimal sample preparation: desalted, concentrated, and digested with trypsin. Equal amounts of peptides were analyzed in duplicate by LC-MS/MS. The obtained raw files were analyzed using Sequest HT algorithm in Proteome Discoverer (Thermo). The parsimony principle was applied and proteins with similar sequences sharing the same identified peptides were reported as one group.

**Figure 1:**
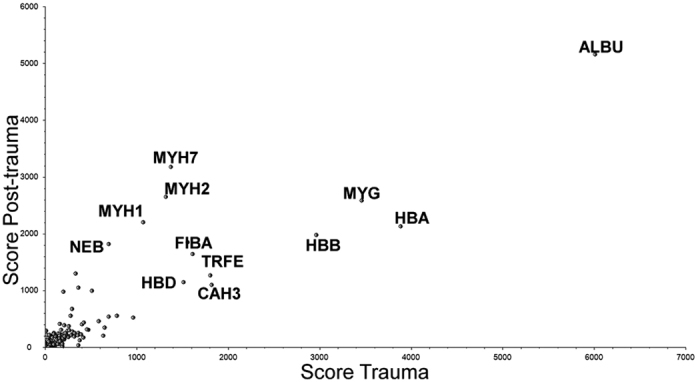
Scatter diagram of the protein changes. Scatter plot of T versus PT protein scores (sum of the scores of individual peptides) of all confidently identified proteins. Each dot in the figure represents one of the proteins.

This straightforward approach allowed the identification of 2516 proteins, in total, merged in to 579 protein groups. A substantial variance between individual samples was observed. Only about 40% of all proteins were identical between all individual subjects from both T and PT periods. Datasets for T and PT samples for proteins detected with top PSMs and identified with the largest number of peptides, were generally the same for all samples (Fig. 1). Proteins identified with top scores were generally the same for T and PT samples (Tables 1 and 2) and includes structural proteins and proteins abundant in plasma/serum. Actin and myosin isoforms are major proteins in muscle cells known to be released into blood in case of muscle damage^13^. Ten proteins (serum albumin, hemoglobin, carbonic anhydrase-1, immunoglobulins, α1-antitrypsin, serotransferrin, fibrinogen, complement C3, hemoplexin, and prothrombin) were identified with top scores in all fractions are well known as the most abundant blood/serum/plasma proteins (Fig. 1).

Apparently, all these highly abundant proteins are present in extracellular space of the muscle as a result of catheter injury and can potentially obscure low-abundant species for proteomic analysis: this problem is well known for many proteomic studies for biomarkers^14^. The excess of serum proteins compared to other proteins was identified as a big technical challenge of MD^15^,^16^,^17^ and proteomic analysis of muscle interstitium can be compromised by the presence of high abundant proteins from blood/serum/plasma. Indeed, a depletion step would decrease sample complexity and improve detection and quantification of extremely low abundant proteins, however there is always the risk to remove some proteins of interest associated with high-abundance proteins. At the same time, many of skeletal muscle enzymes are known as the useful markers of muscle injury. For example, levels of creatine kinase, myoglobin, troponin, and carbonic anhydrase (CAIII) vary widely in both pathological and physiological conditions^18^.

Data files obtained in Proteome Discoverer were exported and further validated in Scaffold software with 90% peptide identification probability, 99% protein identification probability parameters and with minimum 2 peptides required for protein identification, resulting in a 0.0% peptide decoy false discovery rate. Proteins were merged into 212 clusters according to Scaffold’s protein cluster analysis algorithm. (Supplementary data S1).

Scaffold software was used to perform a comparative differential analysis of T and PT samples, employing Student’s t-test using normalized spectral abundance factor method (NSAF) to normalize run-to-run variations^19^. Sixteen proteins were found to have statistically significant differential abundance in T and PT samples (Table 3). Most of these 16 proteins are known to leak out into the blood in case of tissue damage and represent markers of cellular damage. Four proteins related to muscle energy metabolism: isoform 2 of triosephosphate isomerase; phosphoglycerate kinase; creatine kinase and beta-enolase M-type were significantly higher at initial T and decreased in PT fractions.

Creatine kinase plays a central role in energy transduction especially in skeletal muscle tissues, where rapid energy consumption is needed, and creatine kinase levels/activity in plasma are known to vary between healthy subjects depending on age, gender, race, and physical activity^20^. In conditions that damage skeletal muscles or brain (heart attacks, myositis, strenuous exercises, muscular dystrophy, cerebral diseases, any mechanical muscle damage), creatine kinase is released from muscle into the blood. The routine test for elevated levels of creatine kinase in blood is traditionally used to detect inflammation of muscles or serious muscle damage^21^. Phosphoglycerate kinase, triosephosphate isomerase, and muscle specific glycolytic enzyme beta-enolase play important roles in glycolysis and glucogenesis and are essential for efficient energy production. Beta- enolase is also known as a serum marker for muscle damage^22^.

The levels of 12 proteins (myozenin-1, troponins, vimentin, profilin, RhoGDI2, alpha-crystallin B, histone H1.5, S100-A8 and S100-A9) were increased in PT samples in comparison to initial T samples. Four of them were muscle contraction and calcium signal transduction -related proteins: myozenin-1 and troponins. In skeletal muscle, calcium plays a pivotal role in signal transduction and is essential for cellular processes such as excitation-contraction coupling^23^. Troponins (Troponin T, fast skeletal muscle, Troponin I, slow skeletal muscle, Isoform 2 of Troponin T, slow skeletal muscle) are regulatory proteins in skeletal and cardiac muscle. Raised troponin level is used as plasma marker of skeletal muscle damage and particularly as an indication of cardiac muscle cell death.

Proteins involved in actin cytoskeleton regulation (vimentin and profilin): Profilin-1 is one of the most important regulators of F-actin dynamics, regulating many intracellular functions implicated to play a role in many pathological conditions^24^. Vimentin is known to be implicated in the regulation of cell migration and proliferation during the wound healing process^25^,^26^,^27^; desmin and vimentin are markers of regeneration in muscle diseases^28^,^29^,^30^, and up-regulated vimentin level is a marker of skeletal muscle injury^31^.

Among proteins which were up-regulated in all PT samples we identified a two proteins with chaperon activities: Rho GDP**-**dissociation inhibitor 2 (RhoGDI2) and alpha-crystallin B. RhoGDI2 (a member of a small family of chaperone proteins, which controls Rho GTPases^32^) is linked to apoptosis-induced cytoskeletal reorganization and is potentially associated with oxidative stress, apoptosis, and wound healing^33^. Alpha-crystallin B (Heat shock protein beta-5, HspB5) is part of the small heat shock protein family and is able to interact with misfolded proteins to prevent protein aggregation and a wide range of cell stress conditions, as well as inhibit apoptosis and contribute to intracellular architecture^34^. During cell stress, induction and secretion of HSPs leads to pro-inflammatory cytokine and chemokine release activating immune responses^35^. Alpha-crystallin B was found to be upregulated in trapezius muscle of chronic widespread pain subjects in comparison to healthy control group in our previous MD-study^12^.

The level of Histone H1.5 was also increased in PT samples. Beside the classical gene-regulating role, extracellular histones bind to receptors and trigger activation of multiple signaling pathways. Histone levels are known to be significantly elevated in response to injury and involved in the regulation of inflammation^36^. Furthermore, the linker Histone H1.5 was suggested to play a role in the development of muscle^37^,^38^ and may stimulate the proliferation of satellite myoblasts during skeletal muscle regeneration^39^.

Calcium binding proteins S100-A8 and its binding partner S100-A9 (calgranulin A and B respectively) are involved in the regulation of inflammatory, anti-inflammatory, and immune responses. As well as histone H1.5 and HSPs, these proteins have both intracellular (calcium- binding; regulation of microtubule) and extracellular functions. S100A8 and A9 are known to be released from damaged cells in response to stress and are classified as damage associated molecular pattern (DAMP) molecules^40^. Serum protein levels of S100A8/A9 were found to be elevated in many inflammatory diseases and these proteins are known to be involved in the wound healing process^41^.

**Figure 2:**
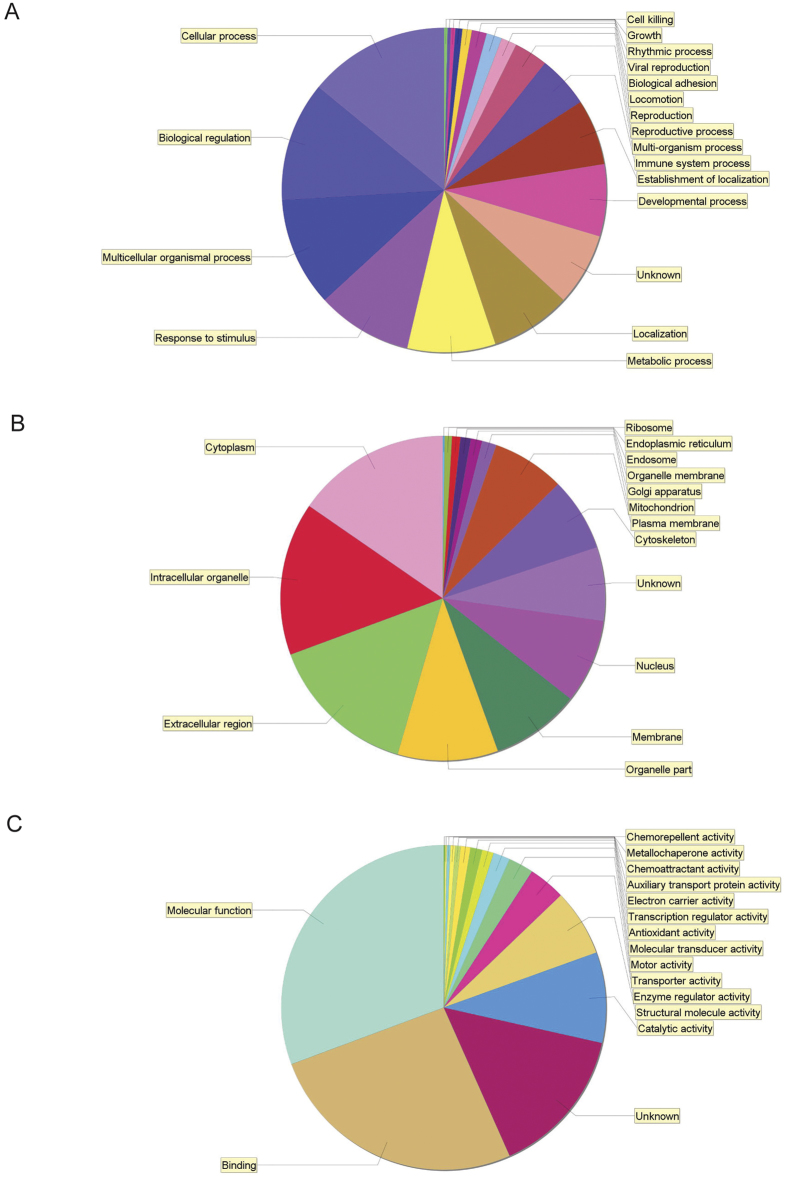
Pie charts classifying the identified proteins according to their biological processes (**A**), cellular components (**B**) and molecular functions (**C**). The identified proteins were grouped according to GO annotations.

**Figure 3:**
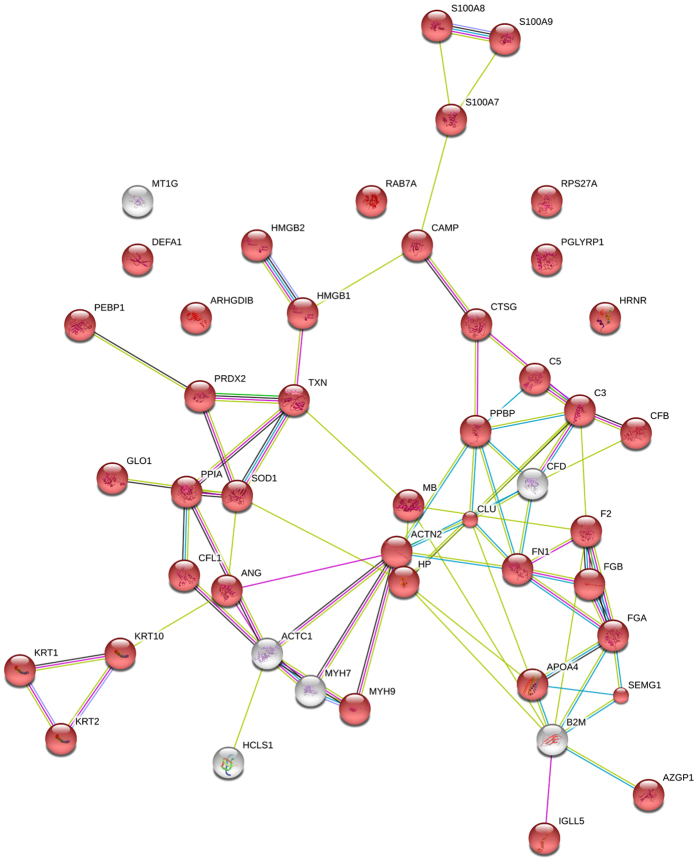
STRING Bioinformatic analysis of the proteins involved in immune system response shown in Table 4. Colored network nodes represent query proteins. Edges represent protein-protein interactions and include different type of actions depicted by the colored lines. For known interactions: pink, experimentally determined; turquoise, from curated databases. For predicted interactions: green, gene neighborhood; blue, gene co-occurrence. For others interactions: olive green, textmining; black, co-expression; purple, protein homology. Extracellular proteins are marked with red color.

To spot possible protein candidates of potential diagnostic value we performed the Scaffold Gene ontology analysis of all identified proteins, which revealed large groups correlated to stimuli response, metabolic process, localization and developmental process (Fig. 2). Fifty-four protein clusters were identified to be involved in immune system processes (Table 4). An interaction map of these proteins was build using String (Search Tool for the Retrieval of Interacting Genes/Proteins, version 10.) (Fig. 3) and according to bioinformatics analysis 41 of 54 inflammatory proteins can be classifies as extracellular region proteins (Fig. 3, red color).

**Figure 4:**
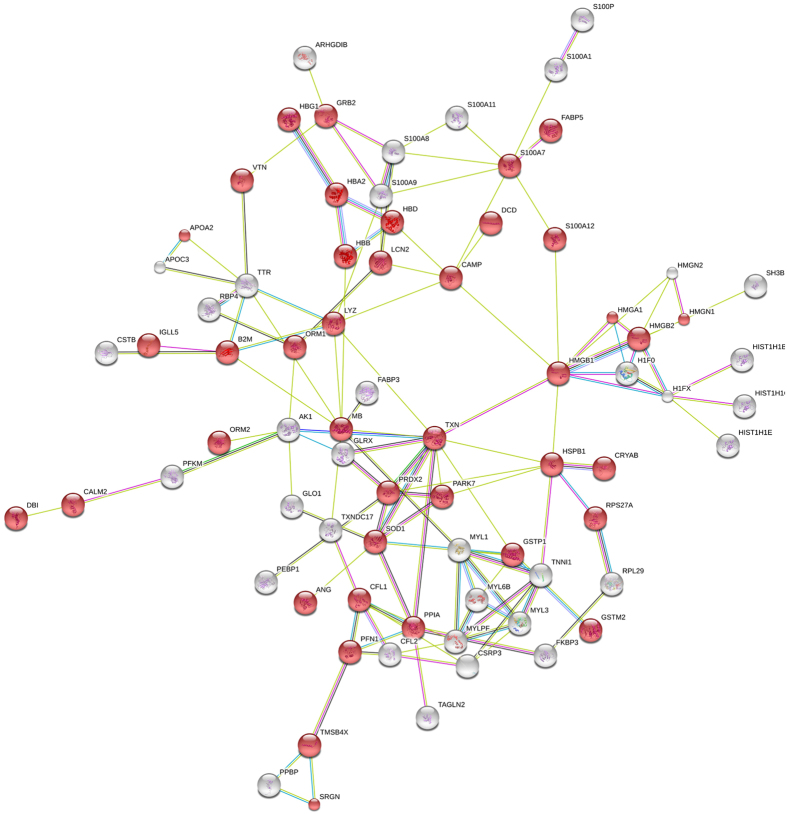
The interaction map of proteins ≤25 kDa (Supplementary S2) obtained by STRING Bioinformatic analysis. Colored network nodes represent query proteins, only the connected nodes are shown, red color indicates extracellular region proteins. Edges represent protein-protein interactions and include different type of actions depicted by the colored lines. For known interactions: pink, experimentally determined; turquoise, from curated databases. For predicted interactions: green, gene neighborhood; blue, gene co-occurrence. For others interactions: olive green, text mining; black, co-expression; purple, protein homology.

Many proteins were annotated to be functionally involved in the regulation of nociception, inflammation and many other essential major biological processes and reported to be potential predictive biomarkers are low-molecular weight proteins (<25 kDa). According to Scaffold analysis, 106 protein groups identified with at least one peptide were corresponding to proteins with molecular weight of less or equal 25 kDa (Supplementary S2). The list of the identified protein was subjected to STRING analysis to reveal functional interactions between the low mass proteins, most of them (as much as 74) belonging to extracellular region proteins. Interestingly, the function of 46 of these proteins was identified as “response to stress” (Fig. 4, red color).

In our previous study we employed the MD technique in combination with 2-D electrophoresis and in-gel digestion to characterize changes in trapezius muscle interstitial proteome in women with chronic myalgia^12^. However, proteins with low-molecular weight are often underrepresented in in-gel digestion based proteomic studies or their coverage and, by that; validity of their identification is small. The limited number tryptic cleavage sites and by that limited number of generated tryptic peptides in the small proteins should be taken into consideration as well.

From the collected fractions we were able to identify 13 proteins involved in nociception (Table 5) with rather low levels and low reproducibility with some exceptions: high molecular weight kininogen, neutrophyl cytosolic factor 2 and calmodulin 5 were identified with 3 or more peptides. Consistent with previous publications^3^,^15^ no significant levels of interleukins were detected in these sampling conditions.

## Conclusions

Proteins involved virtually in stress responses, immune system processes, inflammatory responses and nociception were identified in the interstitial fluid MD proteome in healthy pain free subjects. Moreover, the comparison proteins in “trauma” dialysate collected immediately after catheter insertion with proteins from “post-trauma” MD-fraction revealed 16 differentially abundant proteins (p < 0.05). Our results demonstrate that MD-LC-MS/MS is a promising approach to provide new insights into the interstitial proteome of muscle, and potentially can be further adjusted for biomarker discovery and diagnostics. The presence of highly abundant serum and muscle structural proteins, sample complexity, multiple protein isoforms, protein modifications and low abundance of many proteins of interest are the key challenges. Moreover, careful validation of biological relevance of biomarkers is of great importance for personalized medicine.

The damage caused by the MD probe should be taken into consideration when analyzing disease biomarkers using MD.

## Materials and Methods

### Subjects

Six healthy women (age: 35.1 ± 8.3, BMI: 23.8 ± 2.3) were included in this study. Subjects were instructed not to drink any beverages with caffeine on the day of the study, not to smoke and to avoid NSAID-medication the week before the study. The participants arrived at the clinic in the morning after having eaten breakfast. A brief interview was then made by one of the physicians checking that the instructions had been followed. All subjects reported that they had followed the instructions. During the study, they were not allowed to eat, but they could drink water. All participants gave their informed written consent before the start of the study. The study was approved by the Ethical Committee of Linköping University (Dnr: M10-08) and the methods were carried out in accordance with the approved guidelines.

### Microdialysis

Microdialysis was performed generally as described in ref. 12. Samples were collected during 20–120 minutes after the catheter insertion (trauma period samples, T) and during 140–200 minutes after the catheter insertion (post-trauma samples, PT). All vials were weighed before the experiment started and after each 20 minutes interval in order to confirm that sampling and fluid recovery (FR) was working according to the perfusion rate set. Vials with visible sign of hemolysis were discarded. The samples were stored on ice to prevent protease activation. The samples were then stored as aliquots in −70 °C until analysis.

### Protein extraction and digestion

Samples were subjected to 3 kDa Amicon spin-filter (Merck Millipore) to desalt and concentrate the protein contents. The desalted proteins were dried by speed vacuum concentrator, redissolved in 6 M urea in 25 mM ammonium bicarbonate and incubated at room temperature for at least 30 minutes. The proteins were reduced by incubating in 25 mM DTT for 15 minutes and alkylated with 75 mM iodacetamide for an additional 15 minutes. The samples were diluted 8 times with 25 mM ammonium bicarbonate and filtered by 3 kDa Amicon spin-filter before digestion with trypsin (1:25, w/w trypsin/protein). The digested peptides were dried in speed vacuum concentrator, reconstituted in 0.1% of formic acid in MilliQ water and approximately 0.25 μg was subjected to LC-MS/MS analysis.

### LC-MS/MS analysis

Peptides were separated by reverse phase chromatography on a 20 mm × 100 μm C18 pre column followed by a 100 mm × 75 μm C18 column with particle size 5 μm (NanoSeparatoons, Nieuwkoop, Netherlands) at a fow rate 300 nL/min. EASY-nLC II (Thermo Scientific) by linear gradient of 0.1% formic acid in water (A) and 0.1% formic acid in acetonitrile (B) (0–100% B in 90 min). Automated online analyses were performed with a LTQ Orbitrap Velos Pro hybrid mass spectrometer (Thermo Scientific) with a nano-electrospray source.

### Database searches

Raw files were searched using Sequest HT in Proteome Discoverer (Thermo Fisher Scientific, San Jose, CS, USA; version 1.4.0.288) against a Uniprot Human database (*available at UniProtKB website:* http://www.uniprot.org/taxonomy/9606) with the following parameters: semi trypsin was used as digestion enzyme; maximum number of missed cleavages 2; fragment ion mass tolerance 0.60 Da; parent ion mass tolerance 10.0 ppm; fixed modification- carbamidomethylation of cysteine; variable modifications - N-terminal acetylation. Data were filtered at 1% false discovery rate, high peptide confidence; rank 1 peptides in top scored proteins.

### Data evaluation

Identified proteins were filtered using SCAFFOLD (version 1.4.0.288; Proteome Software Inc., Portland, OR, USA). Identifications were based on a minimum of 2 unique peptides, 90% peptide identification probability (using the Scaffold Local FDR algorithm), and 99% protein identification probability (using the Protein Prophet algorithm), resulting in a 0.0% decoy FDR. Proteins that contained similar peptides and which could not be differentiated based on MS/MS analysis alone were grouped to satisfy the principles of parsimony. The label-free quantitative analysis of peptides was performed by spectral counting analysis, using normalized spectral abundance factor (NSAF), calculated for each protein to normalize run-to-run variations^19^, and quantitative differences were statistically analyzed by a t**-**test. Differences with p values lower than 0.05 were considered statistically significant. Identified proteins were categorized according to gene ontology terms.

String (*Search Tool for the Retrieval of Interacting Genes/Proteins, version 10*) and Pathway Studio (Elsevier) were used for bioinformatics analysis.

## Additional Information

**How to cite this article:** Turkina, M. V. *et al*. Evaluation of dynamic changes in interstitial fluid proteome following microdialysis probe insertion trauma in trapezius muscle of healthy women. *Sci. Rep.* **7**, 43512; doi: 10.1038/srep43512 (2017).

**Publisher's note:** Springer Nature remains neutral with regard to jurisdictional claims in published maps and institutional affiliations.

## Supplementary Material

Supplementary Information

Supplementary Dataset 1

Supplementary Dataset 2

## Figures and Tables

**Table 1:** Top 30 most abundant proteins during the equilibration period.

	Protein ID	Protein name	Sum Xcorr	Sum Coverage, %	Mw, kDa
1	ALBU_HUMAN	Serum albumin	6008,93	90,31	69,3
2	HBA_HUMAN	Hemoglobin subunit alpha	3885,41	87,32	15,2
3	MYG_HUMAN	Myoglobin OS	3458,04	80,52	17,2
4	HBB_HUMAN	Hemoglobin subunit beta	2965,12	95,92	16,0
5	CAH3_HUMAN	Carbonic anhydrase 3	1818,56	88,85	29,5
6	TRFE_HUMAN	Serotransferrin	1807,00	66,91	77,0
7	FIBA_HUMAN	Fibrinogen alpha chain	1611,09	48,38	94,9
8	HBD_HUMAN	Hemoglobin subunit delta	1515,40	93,20	16,0
9	MYH7_HUMAN	Myosin-7	1373,51	28,63	223,0
10	MYH2_HUMAN	Myosin-2	1319,36	27,41	222,9
11	MYH1_HUMAN	Myosin-1	1074,00	23,36	223,0
12	CAH1_HUMAN	Carbonic anhydrase 1	962,34	68,20	28,9
13	A1AT_HUMAN	Alpha-1-antitrypsin	785,71	52,63	46,7
14	FHL1_HUMAN	Isoform 5 of Four and a half LIM domains protein	698,88	71,96	33,6
15	F8WCP0_HUMAN	Nebulin	697,60	17,01	986,1
16	HEMO_HUMAN	Hemopexin	653,26	55,84	51,6
17	TPIS_HUMAN	Isoform 2 of Triosephosphate isomerase	638,12	81,93	26,7
18	PEBP1_HUMAN	Phosphatidylethanolamine-binding protein 1	589,24	75,40	21,0
19	MYH3_HUMAN	Myosin-3	512,92	11,55	223,8
20	FABPH_HUMAN	Fatty acid-binding protein, heart	475,80	63,91	14,8
21	IGHG1_HUMAN	Ig gamma-1 chain C region	457,88	50,91	36,1
22	THRB_HUMAN	Prothrombin	425,90	32,96	70,0
23	HBG1_HUMAN	Hemoglobin subunit gamma-1	421,21	45,58	16,1
24	D6RF35_HUMAN	Vitamin D-binding protein	420,17	50,84	53,0
25	IGKC_HUMAN	Ig kappa chain C region	403,16	82,08	11,6
26	BLVRB_HUMAN	Flavin reductase (NADPH)	395,55	75,73	22,1
27	B7Z7A9_HUMAN	Phosphoglycerate kinase	379,70	57,33	41,4
28	K2C1_HUMAN	Keratin, type II cytoskeletal 1	366,51	39,44	66,0
29	A1AG1_HUMAN	Alpha-1-acid glycoprotein 1	363,39	48,76	23,5
30	KCRM_HUMAN	Creatine kinase M-type	363,20	49,87	43,1

The ranking is based on Peptide Spectrum match (PSM) from high to low.

**Table 2:** Top 30 most abundant proteins during the PT period.

	Protein ID	Protein name	Sum Xcorr	Sum Coverage, %	Mw, kDa
1	ALBU_HUMAN	Serum albumin	5153,68	87,85	69,3
2	MYH7_HUMAN	Myosin-7	3180,15	43,72	223,0
3	MYH2_HUMAN	Myosin-2	2646,74	37,82	222,9
4	MYG_HUMAN	Myoglobin	2582,55	75,32	17,2
5	MYH1_HUMAN	Myosin-1	2200,63	36,10	223,0
6	HBB_HUMAN	Hemoglobin subunit alpha	2132,33	87,32	15,2
7	HBB_HUMAN	Hemoglobin subunit beta	1974,66	89,80	16,0
8	F8WCP0_HUMAN	Nebulin	1815,88	30,92	986,1
9	FIBA_HUMAN	Fibrinogen alpha chain	1640,35	48,27	94,9
10	K1C10_HUMAN	Keratin, type I cytoskeletal 10	1298,61	61,64	58,8
11	TRFE_HUMAN	Serotransferrin	1264,25	72,06	77,0
12	MYH4_HUMAN	Myosin-4	1219,40	24,34	222,9
13	HBD_HUMAN	Hemoglobin subunit delta	1151,19	87,07	16,0
14	CAH3_HUMAN	Carbonic anhydrase 3	1099,53	84,23	29,5
15	K2C1_HUMAN	Keratin, type II cytoskeletal 1	1051,04	57,92	66,0
16	MYH3_HUMAN	Myosin-3	998,14	16,65	223,8
17	K22E_HUMAN	Keratin, type II cytoskeletal 2 epidermal	978,82	74,65	65,4
18	ACTS_HUMAN	Actin, alpha skeletal muscle	675,47	62,86	42,0
19	ACTB_HUMAN	Actin, cytoplasmic 1	558,25	52,53	41,7
20	A1AT_HUMAN	Alpha-1-antitrypsin	558,04	58,13	46,7
21	FHL1_HUMAN	Isoform 5 of Four and a half LIM domains protein 1	536,29	61,82	33,6
22	CAH1_HUMAN	Carbonic anhydrase 1	524,27	68,20	28,9
23	PEBP1_HUMAN	Phosphatidylethanolamine-binding protein 1	463,35	75,40	21,0
24	THRB_HUMAN	Prothrombin	433,85	34,41	70,0
25	CO3_HUMAN	Complement C3	413,23	27,30	187,0
26	IGKC_HUMAN	Ig kappa chain C region	405,29	82,08	11,6
27	GDIR2_HUMAN	Rho GDP-dissociation inhibitor 2	388,45	70,15	23,0
28	K1C9_HUMAN	Keratin, type I cytoskeletal 9	373,16	52,49	62,0
29	HEMO_HUMAN	Hemopexin	350,64	56,28	51,6
30	ALBU_HUMAN	Ig gamma-1 chain C region	318,41	52,42	36,1

The ranking is based on Peptide Spectrum match (PSM) from high to low.

**Table 3:** Proteins significantly (*p* < 0.05) altered between T and PT samples.

Protein	Protein ID	Mw, kDa		Fold change
Protein S100-A9	S10A9_HUMAN	13 kDa	post-trauma high, trauma low	5.5
Protein S100-A8	S10A8_HUMAN	11 kDa		6.3
Profilin-1	PROF1_HUMAN	15 kDa		2.2
Lysozyme C	LYSC_HUMAN	17 kDa		2.5
Rho GDP-dissociation inhibitor 2	GDIR2_HUMAN	23 kDa		3.1
Histone H1.5	H15_HUMAN	23 kDa		3.1
Troponin T, fast skeletal muscle	TNNT3_HUMAN	30 kDa		4.7
Isoform 2 of Troponin T, slow skeletal muscle	TNNT1_HUMAN	30 kDa		7.4
Troponin I, slow skeletal muscle	TNNI1_HUMAN	22 kDa		3.1
Myozenin-1	MYOZ1_HUMAN	32 kDa		1.7
Vimentin	VIME_HUMAN	54 kDa		8.7
Alpha-crystallin B chain	CRYAB_HUMAN	20 kDa		17
Isoform 2 of Triosephosphate isomerase	TPIS_HUMAN	27 kDa	post-trauma low, trauma high	0.3
Cluster of Phosphoglycerate kinase	PGK1_HUMAN	41 kDa		0.2
Creatine kinase M-type	KCRM_HUMAN	43 kDa		0.06
Cluster of Beta-enolase	ENOB_HUMAN	47 kDa		0.1

**Table 4:** Proteins involved in immune system processes.

Identified Proteins and Clusters	Gene name	Protein ID
Myoglobin	MB	MYG_HUMAN
Phosphatidylethanolamine-binding protein 1	PEBP1	PEBP1_HUMAN
Protein S100-A9	S100A9	S10A9_HUMAN
Isoform 2 of Fibrinogen alpha chain	FGA	FIBA_HUMAN
Protein S100-A8	S100A8	S10A8_HUMAN
Superoxide dismutase [Cu-Zn]	SOD1	SODC_HUMAN
Keratin, type II cytoskeletal 1	KRT1	K2C1_HUMAN
Neutrophil defensin 1	DEFA1	DEF1_HUMAN
Rho GDP-dissociation inhibitor 2	ARHGDIB	GDIR2_HUMAN
Keratin, type I cytoskeletal 10	KRT10	K1C10_HUMAN
Peptidoglycan recognition protein 1	PGLYRP1	PGRP1_HUMAN
High mobility group protein B2	HMGB2	HMGB2_HUMAN
Keratin, type II cytoskeletal 2 epidermal	KRT2	K22E_HUMAN
Peptidyl-prolyl cis-trans isomerase A	PPIA	PPIA_HUMAN
Cofilin-1 OS = Homo sapiens	CFL1	COF1_HUMAN
Fibrinogen beta chain	FGB	FIBB_HUMAN
Prothrombin	F2	THRB_HUMAN
Ubiquitin-40S ribosomal protein S27a	RPS27A	RS27A_HUMAN
Actin, alpha cardiac muscle 1	ACTC1	ACTC_HUMAN
Complement C3	C3	CO3_HUMAN
Myosin-9	MYH9	MYH9_HUMAN
Myosin-7	MYH7	MYH7_HUMAN
Ig lambda-2 chain C regions	IGLC2	LAC2_HUMAN
Complement component C4B (Childo blood group	C4B-1	A2BHY4_HUMAN
Metallothionein-1G	MT1G	MT1G_HUMAN
Ig gamma-1 chain C region	IGHG1	IGHG1_HUMAN
Alpha-actinin-2	ACTN2	ACTN2_HUMAN
Haptoglobin	HP	HPT_HUMAN
Ig alpha-1 chain C region	IGHA1	IGHA1_HUMAN
Ig heavy chain V-III region BUT	N/A	HV306_HUMAN
Complement factor B	CFB	B4E1Z4_HUMAN
Ig kappa chain C region	IGKC	IGKC_HUMAN
Zinc-alpha-2-glycoprotein	AZGP1	ZA2G_HUMAN
Ig gamma-2 chain C region	IGHG2	IGHG2_HUMAN
Beta-2-microglobulin	B2M	B2MG_HUMAN
Ig gamma-4 chain C region	IGHG4	IGHG4_HUMAN
Thioredoxin	TXN	THIO_HUMAN
Isoform 2 of Semenogelin-1	SEMG1	SEMG1_HUMAN
Isoform 2 of Clusterin	CLU	CLUS_HUMAN
Apolipoprotein A-IV	APOA4	APOA4_HUMAN
Angiogenin	ANG	ANGI_HUMAN
Cathelicidin antimicrobial peptide	CAMP	CAMP_HUMAN
Cathepsin G	CTSG	CATG_HUMAN
Complement C5	C5	CO5_HUMAN
Complement factor D	CFD	CFAD_HUMAN
Hematopoietic lineage cell-specific protein SV = 3	HCLS1	HCLS1_HUMAN
High mobility group protein B1	HMGB1	HMGB1_HUMAN
Hornerin	HRNR	HORN_HUMAN
Isoform 11 of Fibronectin	FN1	FINC_HUMAN
Lactoylglutathione lyase	GLO1	LGUL_HUMAN
Peroxiredoxin-2	PRDX2	PRDX2_HUMAN
Platelet basic protein	PPBP	CXCL7_HUMAN
Protein S100-A7	S100A7	S10A7_HUMAN
Ras-related protein Rab-7a	RAB7A	RAB7A_HUMAN

**Table 5:** Proteins potentially involved in regulation of nociception pathways.

Gene name	Protein name	No of peptides sequenced	Mw
KNG1	High molecular weight kininogen	16	71.9
NCF2	Neutrophil cytosolic factor 2	4	50.3
CALML5	Calmodulin 5	3	15.9
PKIA	protein kinase inhibitor alpha	1	8.0
RIMS1	Regulating synaptic membrane exocytosis protein 1	1	91.5
PRKX	cAMP-dependent protein kinase catalytic subunit PRKX	1	40.9
PRKD1	Serine/threonine-protein kinase D1	1	101.6
IRS1	Insulin receptor substrate 1	1	131.5
GFRA1	GDNF family receptor alpha-1	1	37.7
NTF3	Neurotrophin-3	1	
PRKD1	Protein kinase D1	1	29.3
KCNC1	Potassium voltage-gated channel subfamily C member 1	1	69.5
CCL14	Chemokine (C-C motif) ligand 14	1	10.7
