# Supplementary material for: Evaluation of dynamic changes in interstitial fluid proteome following microdialysis probe insertion trauma in trapezius muscle of healthy women
Source: Sci Rep. 2017 Mar 7;7:43512. doi: 10.1038/srep43512 (PMC5339898; doi:10.1038/srep43512)
Supplement: Supplementary Information [file srep43512-s1.pdf]

Evaluation of dynamic changes in interstitial fluid proteome following microdialysis in trapezius muscle of healthy women

Maria V. Turkina[1], Nazdar Ghafouri[2], Björn Gerdle[2], Bijar Ghafouri[2]

[1] Division of Cell Biology, Department of Clinical and Experimental Medicine, Linkoping University, Sweden

[2] Pain and Rehabilitation Centre, and Department of Medical and Health Sciences, Linköping University, Linköping, Sweden

Supplementary information

Supplementary S1. Report for proteins identified in microdialysis samples by Scaffold with at least 2 peptides. Protein clusters are expanded and GO annotations are shown.

Supplementary S2. Report for proteins with mass under 25kDa identified in microdialysis samples by Scaffold with at least 1 peptide. Protein clusters are expanded and GO annotations are shown.
